# Effect of Sextant Fixating Angle of Spiral Clavicle Plate on Biomechanical Stability—A Preliminary Finite Element Study

**DOI:** 10.3390/bioengineering11070713

**Published:** 2024-07-13

**Authors:** Ming-Hsien Hu, Po-Feng Su, Kun-Jhih Lin, Wen-Chuan Chen, Shun-Ping Wang

**Affiliations:** 1 Department of Post-Baccalaureate Medicine, College of Medicine, National Chung Hsing University, Taichung 402202, Taiwan; minghsienhu@gmail.com; 2 Department of Orthopedic, Show Chwan Memorial Hospital, Changhua 500009, Taiwan; 3 Department of Orthopedics, Changhua Christian Hospital, Changhua 500006, Taiwan; 88660@cch.org.tw; 4 Technology Translation Center for Medical Device, Chung Yuan Christian University, Taoyuan 320314, Taiwan; kjlin2009@gmail.com (K.-J.L.); wcchen802@gmail.com (W.-C.C.); 5 Department of Orthopedics, Taichung Veterans General Hospital, Taichung 407219, Taiwan

**Keywords:** spiral, clavicle plate, midshaft fracture, finite element analysis, biomechanics

## Abstract

Introduction: A spiral clavicle plate has been accepted for its superior multidirectional compatibility in the treatment of midshaft clavicle fractures from a biomechanical perspective. However, the influence of the sextant angle (spiral level) definition on biomechanical performance has not been clarified. A conceptual finite element analysis was conducted to identify the advantages and drawbacks of spiral clavicle plates with various sextant angle definitions. Methods: Conventional superior and three different conceptual spiral plates with sextant angle definitions ranging from 45 to 135 degrees were constructed to restore an OTA 15-B1.3 midshaft clavicle fracture model. Three major loading scenarios (cantilever downward bending, axial compression, and axial torsion) were simulated to evaluate the reconstructed structural stiffness and the stress on the clavicle plate and bone screws. Results: The spiral clavicle plate demonstrated greater capability in resisting cantilever downward bending with an increase in sextant angle and showed comparable structural stiffness and implant stress compared to the superior clavicle plate. However, weakened resistance to axial compression load was noted for the spiral clavicle plate, with lowered stiffness and increased stress on the clavicle plate and screws as the spiral level increased. Conclusion: The spiral clavicle plate has been reported to offer multidirectional compatibility for the treatment of midshaft clavicle fractures, as well as geometric advantages in anatomical matching and reduced skin prominence after surgery. The current study supports that remarkable cantilever bending strength can be achieved with this plate. However, users must consider the potential drawback of lowered axial compression resistance in safety considerations.

## 1. Introduction

Clavicle midshaft fractures are primarily caused by poor support posture of the upper extremity from falling or by a direct impact on the clavicle [1,2]. Specifically, the clavicle midshaft fracture is the most common trauma of the scapular girdle, occurring in about up to 81% of all clavicle fractures [3,4]. Conventionally, non-displaced clavicle fractures are managed with conservative treatment [5,6]. However, a higher occurrence of displacement (48%) and comminuted (19%) patterns in clavicle midshaft fractures has been reported [7]. Several comparative studies have revealed poorer clinical outcomes from non-operative treatment of clavicle midshaft fractures compared to surgical treatment [8,9,10,11]. Recent advancements in techniques and devices have introduced various surgical treatments for clavicle midshaft fractures. While non-operative approaches were once common, surgical options like interfragmentary screw fixation, intramedullary fixation, cerclage wiring, and plate fixation are now preferred for displaced midshaft fractures [12,13,14], yielding better outcomes that reduce non-union and malunion rates compared to conservative treatments [15,16]. In addition, high-end technology using additive manufacturing to reproduce fracture patterns offers a precise three-dimensional reference for the trauma field, promoting ideal techniques in surgical planning and implant design [17].

**Figure 1:**
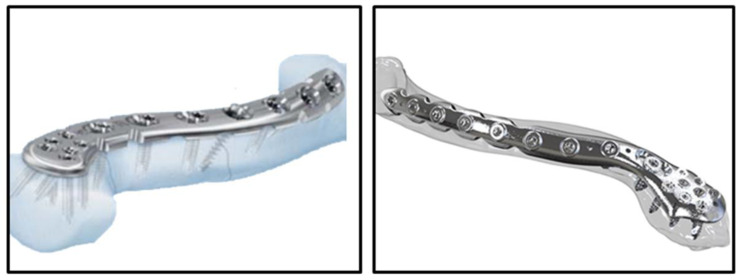
Commercialized spiral clavicle plate: (**left**) SYNTHES LCP superior anterior clavicle plate and (**right**) Aplus C.A.S. locking plate system.

The general geometry of a bony shaft plate is straight or slightly curved to fit the anatomical features. To address concerns about multiple segmental fixations and improve screw pull-out strength, spiral (or helical) bone plate fixation has been introduced in previous studies [18,19,20]. The concept of the spiral clavicle plate has been developed over the past two decades and is now commercially available (Figure 1). Previous clinical studies have reported high postoperative satisfaction [21], alongside demonstrated biomechanical benefits in both experimental and computational studies. These include enhanced bending stiffness, rotational rigidity, prevention of neurovascular damage, and reduced prominence of hardware [22,23,24,25]. With a surgical technique similar to conventional clavicle plate implantation, the enhancement in screw pull-out strength by spiral plate fixation can be predicted based on screw orientation, as demonstrated in previous studies of helical proximal humerus plate design [20].

For a more thorough evaluation of implant safety, it is essential to understand the effect of the spiral feature of the clavicle plate on detailed biomechanical responses. Additionally, it is unclear whether the spiral angle of the clavicle plate or the sextant angle of the screw fixation would also result in significant differences in structural behavior. The current study aims to conduct an objective biomechanical comparison among three different conceptual spiral features of clavicle plate designs and the conventional superior clavicle plate using the finite element method. The study evaluated the multi-directional stabilizing effect and implant stresses of different clavicle plate designs in the treatment of OTA 15-B1.3 midshaft transverse fractures.

## 2. Materials and Methods

**Figure 2:**
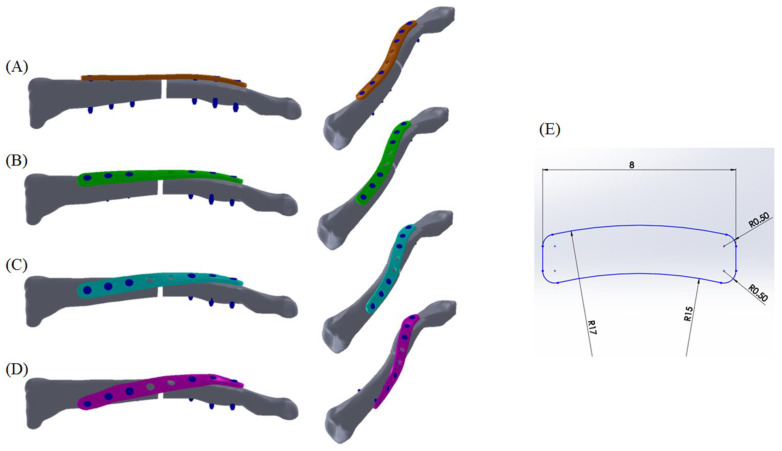
Conceptual clavicle plate models: (**A**) SP-0 model, (**B**) SP-45 model, (**C**) SP-90 model, (**D**) SP-135 model, and (**E**) same cross-sectional pattern in all plate models.

**Figure 3:**
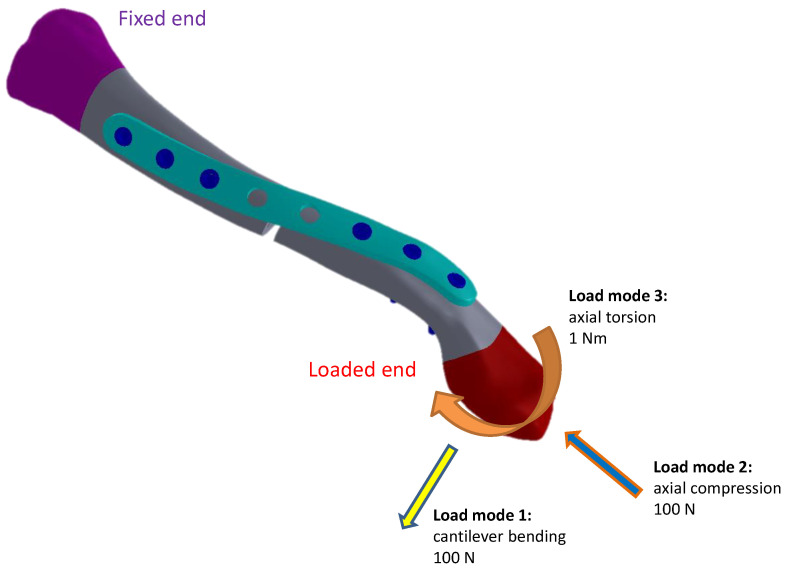
Fixed boundary and the 3 loading conditions (cantilever downward bending, axial compression, and axial torsion) applied in the current study.

**Figure 4:**
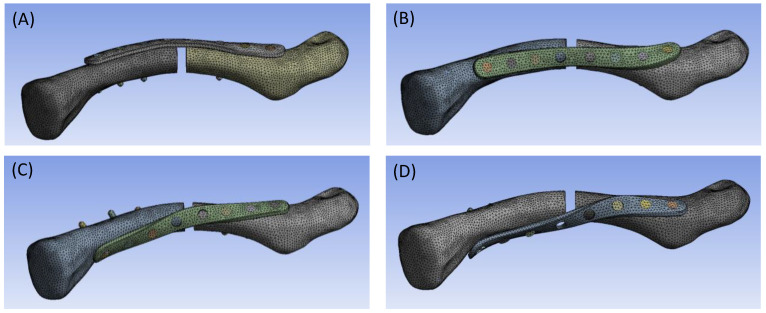
Meshed finite element models applied in the current study: (**A**) SP-0, (**B**) SP-45, (**C**) SP-90, and (**D**) SP-135.

A three-dimensional clavicle bone model was reconstructed in commercial software Amira 3.1 using computed tomography images of a healthy 69-year-old male (left clavicle, slice thickness: 1.25 mm, 512 × 512 pixels per image). A 2.5 mm fracture gap was created at the midshaft location [26] to simulate the OTA 15-B1.3 transverse clavicle fracture, as confirmed by experienced surgeons. Clavicle plate models, including (1) the superior plate (SP-0, Figure 2A), (2) the 45° spiral clavicle plate (SP-45, Figure 2B), (3) the 90° spiral clavicle plate (SP-90, Figure 2C), and (4) the 135° spiral clavicle plate (SP-135, Figure 2D), were constructed with identical cross-sectional dimensions (2 mm thickness and 8 mm width, Figure 2E). All plate models were standardized to a length of 80 mm parallel to the clavicle axis, with their curvatures matching the contour of the clavicle bone surface as possible. The spiral increment parameters were 0°/mm for the SP-0 model, 0.5625°/mm for the SP-45 model, 1.125°/mm for the SP-90 model, and 1.6875°/mm for the SP-135 model. Each clavicle plate model was equipped with 8 screw holes, each measuring 4.0 mm in diameter and spaced 10 mm apart. The axes of all screw holes are perpendicular to their respective plates. In each model, six locking screws with a diameter of 2.4 mm were inserted into the screw holes using bicortical fixation to provide robust fixation [27,28], while the two central screw holes were left empty. To represent screw performance, the screw order was defined from 1 (most distal) to 6 (most proximal). Sextant angles measured from the most proximal to the most distal screw were 0° (all parallel), 39.38°, 78.75°, and 118.13° in the SP-0, SP-45, SP-90, and SP-135 models, respectively.

The screw threads were simplified and designed to bond to both the clavicle bone and the clavicle plate interfaces. To prevent the bone plate from penetrating into the clavicle, frictionless contact was assigned to the plate/bone interface [29,30]. A frictional coefficient of 0.2 was assigned to both ends of the bone fragments that form the central clavicle bone fragment gap, to address potential contact resulting from loading [29,30]. All target–contact pair definitions are listed in Table 1. Material properties for the cortical bone, cancellous bone, and titanium alloy were assigned accordingly (Table 2) [29,30,31]. The models were fixed at the proximal end of the clavicle, while three different loading modes were respectively assigned for multi-directional biomechanical evaluation of the different clavicle plate design models: (1) a 100 N cantilever bending load (downward); (2) a 100 N axial compressive load; and (3) a 1 Nm axial torque, as shown in Figure 3 [29,30,32]. Convergence tests were conducted to ensure the appropriate 10-node tetrahedral elements contained in SP-0, SP-45, SP-90, and SP-135 models, respectively, numbering 157,396, 155,471, 152,157, and 154,237 in commercial software Ansys Workbench 17.1 (Figure 4).

The structural stiffness under bending and axial compression loads was determined by evaluating the displacement of the most distal locking screw in each model. Additionally, the rotation angle in axial torsion was evaluated by measuring the change in screw axis angle between the most proximal and most distal screws before and after loading. A comparison based on von Mises stress was conducted to evaluate the potential risk of implant failure for all clavicle plate and screw models.

## 3. Results

### 3.1. Structural Stiffness

**Figure 5:**
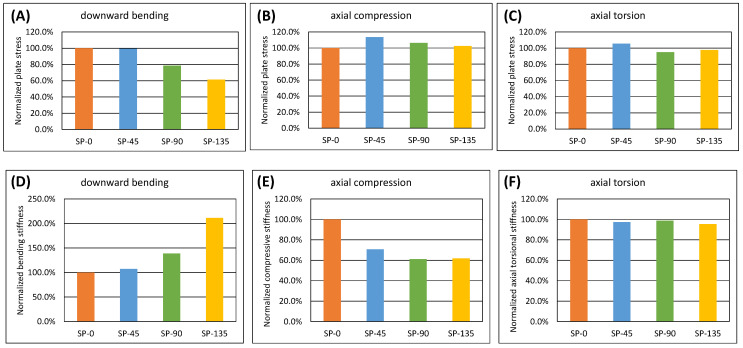
Simulated results normalized by SP-0 model: (**A**–**C**) comparisons of plate stress under various loading modes and (**D**–**F**) comparisons of structural stiffness under various loading modes.

In all simulated models observed in the current study, there was no interfragmentary contact under the applied loading conditions. After normalizing the linear and torsional structural stiffness based on the SP-0 model data (downward bending stiffness: 10.9 N/mm; axial compressive stiffness: 163.7 N/mm; axial torsional stiffness: 0.244 N/°), all spiral clavicle plate models showed increased stiffness under downward bending (SP-45: +7.1%; SP-90: +38.6%; SP-135: +111.4%), but lower stiffness under axial compression (SP-45: −29.4%; SP-90: −38.9%; SP-135: −38.1%). Minor differences were observed among all models under axial torsion (within ±5%) (Figure 5A–C).

### 3.2. Stress on Clavicle Plates

**Figure 6:**
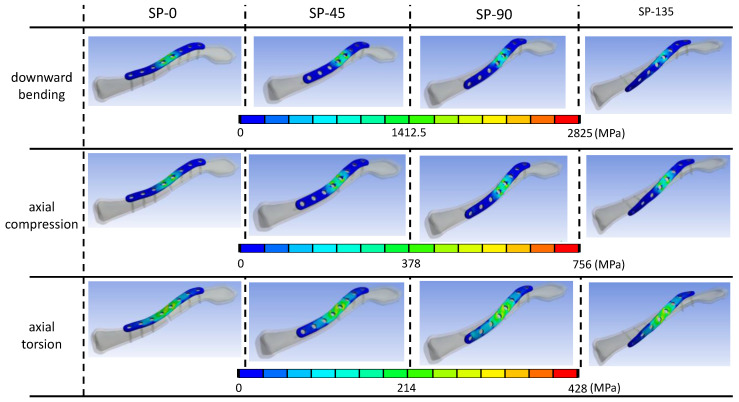
The maximal von Mises stress located on the clavicle plate models.

In all simulated models across various loading conditions, stress concentration was found on the clavicle plates, particularly around the two central screw holes adjacent to the fracture site where the screw holes were left empty (Figure 6). Under cantilever bending load, the highest von Mises stress was found in the SP-0 model (2825 MPa), decreasing with the increase in the sextant angle of screw fixation (SP-45: 2804 MPa; SP-90: 2220 MPa; SP-135: 1735 MPa). Under axial compression load, the SP-45 model exhibited relatively higher stress (775.9 MPa) compared to SP-90 (707.0 MPa), SP-135 (681.7 MPa), and SP-0 models (665.0 MPa). In axial torsion loading condition, the SP-45 model showed the highest stress (428.6 MPa), followed by SP-0 (405.7 MPa), SP-135 (396.4 MPa), and SP-90 models (385.4 MPa). Figure 5D–F illustrates a numerical comparison normalized based on the stress magnitude of the superior plate model.

### 3.3. Stress on Locking Screws

**Figure 7:**
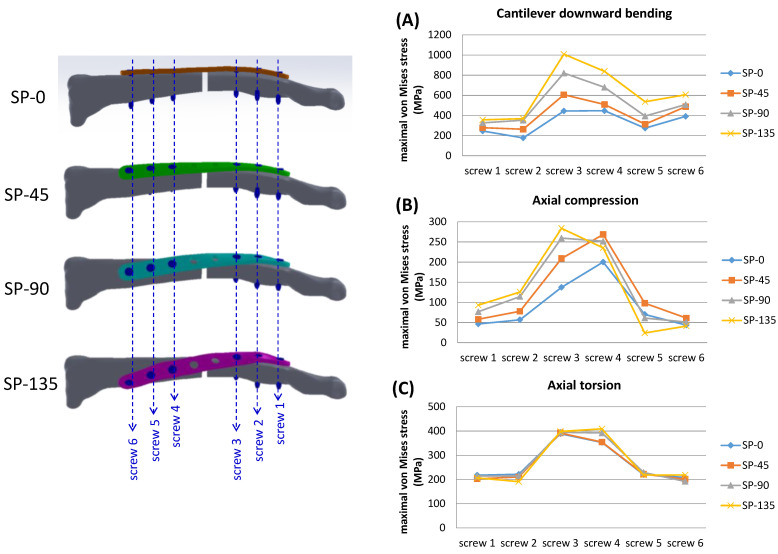
Definition of screw locations and the maximal von Mises stress located on the locking screws in each loading condition: (**A**) cantilever downward bending, (**B**) axial rotation, and (**C**) axial torsion.

Elevated stress levels were observed beneath the screw head for every locking screw across all models, with the greatest stresses found on screws adjacent to the designated fracture gap in each model. Screw stresses in spiral clavicle models were generally higher compared to the SP-0 model (Table 3), especially for the center two screws (screws 3 and 4). Differences in screw stress were relatively minor in the axial torsion loading mode (Figure 7).

## 4. Discussion

For a better understanding of the geometrical effect of the spiral clavicle plate, finite element analyses have been conducted to compare the quantified performances with those of a conventional superior clavicle plate in the current study. The main findings in the current simulations were that an increased sextant angle (i.e., the spiral level) of the clavicle plate resulted in greater resistance to cantilever bending load, similar strength in axial torsion, but lower structural stiffness in axial compression. The multi-directional loading conditions in the current study revealed elevated average screw stress with the increase in sextant angle of the clavicle plate under cantilever bending and axial compressive loads. Users who believe in the benefits brought by the spiral clavicle plate in the treatment of clavicle midshaft fractures must keep the biomechanical safety issues in mind. The findings presented in this study could enhance orthopedic surgeons’ understanding and confidence regarding the future application of spiral clavicle plates in clinical practice.

The open-reduction internal fixation technique has been accepted for treating clavicle fractures, supported by clinical evidence indicating reduced operation time, improved fracture healing efficacy, and decreased medical expenses [9,10,11]. Several clinical studies have demonstrated that complications, including malunion, non-union, structural instability, and shortening of fracture sites [8], can occur after conservative treatment of midshaft clavicle fractures. Although good long-term outcomes of clavicle midshaft fractures treated conservatively have been reported in some studies, surgical fixation is necessary specifically for displaced clavicle midshaft fractures to reduce the risk of non-union [33,34].

### 4.1. Structural Stiffness

Maintaining adequate structural stability after anatomical reduction through clavicle plate fixation is essential for a better clinical outcome. Previous biomechanical studies, both experimental [35] and computational [26], have shown that for the fixation of midshaft clavicle fractures, the superior plating technique provides increased structural rigidity in axial compression and torsional modes compared to the anterior plating technique. In another biomechanical study conducted by Partal et al. [24], 3.5 mm pelvic reconstruction plates were used to compare superior and anterior fixations for midshaft clavicle fractures. While their findings for axial compression were consistent with those of Iannotti et al. [35], Partal et al. reported greater bending rigidity and torsional stiffness with the anterior fixation technique [24]. To achieve better multidirectional compatibility, the spiral clavicle plate, also known as the superoanterior clavicle plate, is gaining popularity for treating displaced midshaft clavicle fractures. In the biomechanical study by Eden et al., the 7-hole superoanterior clavicle locking compression plate demonstrated superior structural stability in the reconstruction of midshaft clavicle fractures compared to the 7-hole reconstruction plate [22]. Marie’s finite element study also concluded that the anatomically shaped superoanterior clavicle plate is more stress-resistant and stable compared to a regular reconstruction plate [23]. The biomechanical study conducted by Zhang et al. [32] analyzed the efficacy of treating midshaft clavicle fractures using either a spiral plate or a Herbert screw, concluding that spiral plate fixation provides better mechanical stability and is more suitable for patients requiring early return to activity. In the current study, the downward cantilever bending stiffness increased with the increasing spiral angle of the clavicle plate model (bending stiffness: SP-135 > SP-90 > SP-45 > SP-0). This improvement is attributed to the spiral geometry, which partially reveals the biomechanical characteristics of the anterior clavicle plate, especially at the location where the plate spans the fracture line. It can be expected that a spiral clavicle plate with a larger spiral angle will exhibit even greater strength because the plate can be positioned anteriorly to the fracture line, but this may lead to challenges in achieving anatomical fit and during surgical operations. On the other hand, the greatest axial compressive stiffness observed in the SP-0 model partially echoes previous studies [20,24,26], as the spiral clavicle plate designs in the current study serve as transitional forms between a superior clavicle plate and an anterior clavicle plate. The reduced support of the plate structure along the axis parallel to the direction of load application weakens its resistance to the load. Good callus formation in the secondary healing phase after fracture reduction can be expected if the contact between fractured segments is balanced in the axial direction. Insufficient bending rigidity of the bone plate system may result in the tilting of the structure rather than an ideal axial movement of fracture segments, and callus formation may be concentrated at the cortex opposite the bone plate. Greater bending rigidity in the spiral clavicle plate model may help prevent unbalanced contact with the far cortex, while reduced stiffness in the axial direction could enhance mechanical stimulation for bone healing at the fracture site. The performances in axial torsion mode were similar, which is expected because the material volume participating in resisting the twist of the structure is also quite similar among all simulated clavicle plate models. Additionally, the extra interfragmentary screw can probably aid in fractured fragment reduction and reinforce the biomechanical properties of the structure, but failure at the interfragmentary screw through a horizontal split fracture would be common, especially with superior plating, as reported by Ziegler et al. [36]. The dual plating technique can be another consideration for reinforcing the structural stability by using two smaller constructs to replace the single, large plate fixation, but also raised the problem that more defects are generated when removing the bone screws in removal surgery, jeopardizing the structural integrity [37,38].

### 4.2. Stress on Clavicle Plates

The assessment of implant stress using finite element analysis serves as a valuable tool for safety evaluation. Farve et al. [26] explained that increased stress is expected in the superior and anterior plates under inferior bending load and axial compression load, respectively. Marie’s finite element investigation applied complex loads from daily activities to assess the influence of plate design on plate stress performance. The study revealed that larger stresses are generated on the anteroposterior clavicle model than on traditional superior and anterior plates, whether or not the interfragmentary lag screw was inserted [15]. The current study showed lower plate stresses in all spiral plate models under cantilever bending load and greater stresses compared to the superior clavicle plate model, while the performances under axial torsion mode were similar across all plate designs. In prior finite element investigations, stress concentrations were found at features with specific functions, such as decompression concaves, combi-holes, or reconstruction notches (which aid surgeons in bending the plate to the desired contour) [23,26]. To mitigate potential errors in this stress-based evaluation, the current study standardized the cross-sectional features across all plate models. Stress was found to concentrate around the central two screw holes adjacent to the fracture site in all simulated clavicle plate models. The spiral structure of the clavicle plate may be helpful in supporting against cantilever bending loads, as the region of the plate bridging the fractured segment in spiral clavicle models is located anteriorly and offers a greater moment of inertia compared to the superiorly located clavicle plate model. However, the spring-like performance under axial load could potentially lead to greater structural distortion and increased stress on the plate. The difference in plate stresses under axial torque, similar to the aforementioned performance in terms of torsional stiffness from a mechanical perspective, was comparatively minor compared to those observed under cantilever bending and axial compression loads. To address the issue of high-stress concentration in central clavicle plate fixation for midshaft fractures, several ideas have been proposed, such as modifying plate designs to remove screw holes above the fracture zone [39] or eliminating decompression/low-profile notches near the fracture site [40] to achieve better stress distribution. The other possible way for plate structural reinforcement is to fill the existing, unused screw hole above the fracture site by using a threaded screw head/cap. Bellapianta et al.’s biomechanical study showed a fourfold improvement in fatigue life using Synthes plate and screw head for filling the central, unused screw hole [41]. All the aforementioned strategies are worth considering for future design improvements or reinforcement accessories offered by manufacturers.

### 4.3. Stress on Locking Screws

Previous studies have rarely addressed screw stress in simulated clavicle plate fixation models. In studies on clavicle fracture reconstruction, the focus typically centers on plate failure rather than screw failure. This emphasis on plate failure may be due to its potential implications for the overall stability of the construct. However, in the current study, stress concentration was noted beneath the head of each screw. Due to the lack of structural continuity in the simulated models, screws adjacent to the midshaft fracture gap experienced the most significant deformation. In all simulated models, screws 3 and 4 consistently exhibited the highest stress levels across all loading scenarios. Furthermore, the stress on screws 3 and 4 in spiral clavicle plate models was found to exceed that in the SP-0 model under axial compression load, with stress levels increasing as the sextant fixation angle of the clavicle plate models became greater. A similar finding has been revealed previously, indicating that parallel screw arrangements in the superior clavicle plate provide co-planar-like support to bending forces [30]. On the contrary, the divergent screw arrangement and the spiral feature of the plate could potentially induce structural distortion, particularly affecting the center two screws (adjacent to the fracture gap) under more complex mechanical conditions such as shear force and torsion. Some studies recommend the use of bicortical fixation in clavicle plate fixation for midshaft fractures, which offers better bone purchase and stability, to help reduce screw-related complications by maximizing the contact between bone and screw [27,28].

### 4.4. Study Limitations

Several limitations should be noted regarding the study design, modeling, and simulation:

- As a conceptual study in orthopedic biomechanics, the current study conducted finite element analyses using assumed homogeneous, isotropic, and linear elastic material properties. The quantified information obtained may provide guidance for predicting the biomechanical performance of various designs. However, to validate the results, practical biomechanical tests should be considered in future studies.
- In the current study, common functional features found in commercial bone plates, such as decompression concaves, combi-holes, or reconstruction notches, were not considered. Instead, an identical cross-sectional pattern was assigned to all conceptual clavicle plate models to simplify and eliminate potential misjudgment of stress-based results due to complicated geometric designs.
- While screw and plate stress performance may be associated with important aspects of clavicle bone remodeling, the current study focused on the effect of plate geometry on structural performance. Previous biomechanical studies have emphasized the stress-shielding effect after clavicle plate fixation in the treatment of clavicle fractures. Cortical bone atrophy underneath the strong metallic plate may occur [42]. For the newly formed bony structure at the previous fracture site, the bone quality is not as strong as that of the surrounding bone, which is a major cause of re-fracture after clavicle plate removal [17]. Zhang et al.’s finite element study represented end-to-end stress transmission (from the proximal screw hole to the sternal end of the clavicle) through the plate in the reconstructed clavicle model from a midshaft fracture, reflecting an obvious stress-shielding effect after spiral clavicle plate implantation [32]. However, due to simplified finite element definitions and the omission of detailed parameters such as screw thread characteristics, bony trabecular structure, bone density reconstruction from the radiographs, and comprehensive element behavior, the current study may not provide sufficient information on the impact of implant design on bone remodeling and possible stress-shielding performance under appropriate physiological loading conditions.
- The parameters of the clavicle plate design, including plate thickness, width, length, curvature radius, spiral sextant angle, and screw diameters in the current study, are intended solely for conceptual purposes.
- The loading conditions referenced in previous publications [29,30,32] have been applied in the current study. Biomechanical behavior and the influence of implant design can be evaluated for quantitative comparison. However, physiological clavicular activities involve much greater complexity, requiring detailed definitions of soft tissue attachments and consideration of multiple load exertions. The current bony model lacks the scapular part, which is essential for defining appropriate soft tissue origin/insertion boundaries and muscle forces. Nevertheless, the simplified loading conditions introduced in previous studies provide a straightforward viewpoint for biomechanical evaluation.
- The bony model was reconstructed from only one subject. For a more objective evaluation, future studies should consider expanding the research to include different clavicle geometries, bone qualities, and other individual differences.

## 5. Conclusions

As an intermediate concept bridging superior and anterior clavicle plate designs, the spiral clavicle plate models demonstrate better downward cantilever bending performance with increasing sextant angles of plate designs and show a comparable response to axial rotation load compared to the superior plating model. However, average screw stress increases with the increasing sextant angle of the clavicle plate. When considering the advantages of the spiral clavicle plate, for safety reasons, users must be cautious of its reduced stiffness under axial compressive loads. Further mechanical tests for biomechanical validation and related experiences in clinical use shall be beneficial to enhance the confidence of practical application.

## Figures and Tables

**Table 1:** Target–contact definition of solid models in the current study [29,30].

Interface	Contact	Target	Behavior	Frictional Coefficient
Plate/screw	plate	screw	bond	-
Plate/bone	bone	plate	contact	Frictionless
Screw/bone	bone	screw	bond	-
Bone/bone	Proximal fragment	Distal fragment	contact	0.2

**Table 2:** Assigned material properties in the current finite element study [29,30,32].

Materials	Young’s Modulus (MPa)	Poisson’s Ratio
Cortical bone	11,000	0.3
Cancellous bone	500	0.1
Titanium alloy	110,000	0.3

**Table 3:** Comparison of maximal, minimal, and average screw stress values in all simulated models (unit: MPa).

	Downward Bending			Axial Compression			Axial Torsion		
	Max.	Min.	Avg.	Max.	Min.	Avg.	Max.	Min.	Avg.
SP-0	447.6	177.1	330.1	199.9	43.7	92.4	389.3	208.5	268.4
SP-45	606.0	262.8	409.5	268.5	58.1	128.7	392.2	201.2	263.9
SP-90	820.5	326.5	513.7	259.4	51.1	135.7	394.7	192.1	272.8
SP-135	1008.2	356.6	619.1	283.7	24.1	133.9	408.5	191.0	273.4

## Data Availability

All data have been revealed in the manuscript.
